# Progress in Medicine

**Published:** 1895-07-20

**Authors:** 


### Progress in Medicine.

### DISEASES OF THE RESPIRATORY SYSTEM.

(*Continued.*)

**Tubercular Meningitis.**—A case of recovery has been reported by Freyhan where the diagnosis was confirmed by puncture and bacilli found in the fluid.[1] Another was brought forward by S. West. Here two children of the same family were attacked with similar symptoms, one died and showed post-mortem evidence of tubercle, while the other gradually recovered.[2] The evidence here was strong, but of course not absolutely convincing. Dennig insists on the uncertainty of all symptoms distinctive from so-called simple meningitis. In a case he mentions where pneumo-coccal meningitis appeared to be present, true tubercle bacilli were found. The value of lumbar puncture and examination of the fluid during life is of great importance.[3] Maher regards the disease as almost always a secondary lesion, and quotes Simon of Nancy as arguing to that effect, except in the rare cases where the bacilli gain entrance through an injury to the ethmoidal foramina. It is impossible to say what causes the tubercular masses forming the primary lesion in the body to send out their colonies to the meninges. Sometimes an injury would seem to be sufficient, as when a child receives a blow on the head. The original entrance to the body has usually occurred through the air that enters the lungs or the food that goes through the stomach. Heredity, as a rule, only accounts for a favourable type of cell for the development of the disease in case of infection, for the bacilli are very rarely transmitted direct from the parents.[4] The treatment of meningitis, therefore, depends on our preventing and curing the primary tuberculosis in the body. It would seem hopeless to attack it directly, though surgical intervention may postpone the fatal ending. [And has effected at least one cure.—ED.] The disease has even followed extirpation of tubercular pyosalpinx, an instance of which is given in detail by Binaud. There were no other centres of infection in this case elsewhere in the body, but frequently tubercle appears after these operations in the lungs and other localities.[5]

**Tubercular Ascites.**—Löhlein has treated eighteen cases by an abdominal incision and removal of the fluid. Six of the eighteen remained permanently cured, and no difference was observed between those cases in which drainage after the operation was employed and those in which there was none.[6] Follet injected air into the abdominal cavity with success, and Von Moorhof into the scrotum, after the withdrawal of the fluid through a canal.[7] In the dry forms of peritoneal tuberculosis the results of operations are not so favourable. Morris has endeavoured to find the reason for the recoveries after operation.[8] He isolated a toxalbumen formed by putrefactive bacteria in the fluid withdrawn in these cases, and discovered that this toxalbumen promptly destroyed tubercle bacilli growing in culture tubes. Moreover, the cases where he operated with complete asepsis were the ones which were not cured, while those succeeded where the operation was less completely guarded, and air entered freely. Hence he considered that the tubercle bacillus was destroyed by the putrefactive organisms introduced during operation.

**Ætiology of Tubercle.**—Sir James Paget raised the question, What is the force of heredity when one parent is a member of a cancerous and the other of a tubercular family? We know that acute tuberculosis and acute cancer never make rapid progress together, but what comes of it when they are mingled by inheritance?[10] W. Roger Williams replied that the evidence he had collected showed that pulmonary tubercle is by far the most prevalent disease among the relatives of cancerous persons. They are much more prone to it than the rest of the community.[11] T. E. Squire, after analysing 1,000 cases of phthisis, came to the conclusion that the influence of heredity in the children of phthisical parents is not shown in more than 9 per cent. over the cases in children of non-phthisical parents. Indeed, the influence is not a true heredity, but a tendency to disease of various kinds from which the children of all weakly parents suffer. J. Hutchinson pointed out in connection with this, that it was very difficult to define phthisical parents, since the disease often remained latent for a great length of time under favourable conditions. The fact of some hereditary influence existing was shown by other speakers from the preferential limitation of the descent to one sex as in recognised hereditary diseases, and from the inheritance of special types of the disease, such as fibroid phthisis, as well as from the particular age at which all members of phthisical families succumb in many cases. Instances, too, of tuberculosis *in utero* have been discovered. Still, as Dr. Heron suggested, much evidence might be adduced that scarlet fever and measles are hereditary in a certain sense, and special types of them are inherited. The slightly greater liability of the children of phthisical parents may be due, indeed, to their exposure to infection in their early days.[12] However, an error in Dr. J. E. Squire's tables has been pointed out by J. K. Fowler, in that he works back from a consumptive in every case, whereas in many families there is no person affected. This would make the influence of heredity higher than 9 per cent.,[13] as the supporters of heredity claim it is.

Besides infection and heredity, certain neuroses, lesions, and poisons of the nerves have a great share in the causation of phthisis. Numerous facts in support of this view have been adduced by T. J. Mays. Chronic poisoning from lead, mercury, and alcohol, the virus of syphilis, whooping cough, influenza, diabetes, and epidemic meningitis act through the nervous system on the lungs, while the direct neuroses existing in epilepsy, asthma, hysteria, and insanity are followed by tuberculosis in an unexpectedly large proportion of cases. Indeed, he would say that any agent which interferes with the integrity of the nervous system has also the power of producing pulmonary phthisis. The converse theorem that idiocy and other nerve lesions are largely due to the influence of the virus of phthisis through heredity is perhaps equally remarkable. Lancereaux[14] has discussed the effect of the poisons of alcohol and absinthe in the causation of phthisis through the nerves. Without these and other predisposing causes the action of the bacillus would, he thinks, be nil. Drunkard's phthisis is usually localised at the right apex and posteriorly, contrary to what is usually taught.[15] S. G. Dixon has carried a step further the question of the production of a tuberculous diathesis by suggesting its dependence on deficient nitrogenous metabolism. He found that the injection of certain amides, creatin, urea, and thiosinamin produced similar results to those following tuberculin, and he deduces from Vaughan's experiments that the active principle of tuberculin is also a xanthin body, plus nucleinic acid, which explains their common reaction in tuberculous subjects, and their power of checking tubercular progress. Moreover, in lithæmic states there is rarely seen any tubercular growth, and the value of the common remedies, cod liver oil and fats, may depend on their action in an allied direction.[16] In short, where there is great nitrogenous metabolism, or much formation and destruction of white corpuscles with their nuclein and uric acid derivatives, tubercle cannot flourish.

In reference to direct heredity Lehman has recorded a fresh instance where a child born of a moribund tubercular mother was found to have tubercle widely disseminated at birth.[17] Schmorl and Kockel have related three others in which the fœtal placental villi were affected, and in one of them bacilli were found in the body of the fœtus.[18] While the placenta to some extent hinders the spread of bacilli, it is not infrequently the seat of disease itself. The passage through the placenta is thus not direct, but a process of "growing through,"[19] the direct conveyance being prevented by the chorionic epithelium and in other ways.

**Treatment of Phthisis.**—Landerer and Moschowitz have injected a 5 per cent. emulsion of cinnamic acid in a large number of cases, which produces leucocytosis, and finally cicatrization of tubercular foci. The former obtained good results, except in advanced and acute cases. Moschowitz had only modorate success, but thinks the method worthy of further trials, while Mader found it entirely useless, and sometimes accompanied by untoward results.[20] The inhalation of oil of peppermint has been recommended by Michele and Carasso. The former employed it by inhalation frequently during the day and night, together with the internal use of creosote,[21] and Carasso appears to find continuous inhalation necessary together with forcible deep inspiration at intervals. A useful method of disinfecting tubercular sputa has been brought forward by Goviansky,[22] which consists in mixing them with wood vinegar or pyroligneous acid. The common antiseptics have but little effect, while wood vinegar completely destroyed all bacilli in spittoons or other receptacles in six hours with certainty.[23]

[1] Am. J. Med. Sc., April, 1895. [2] Lanc., June 1. [3] B. M. J., Jan. 5. [4] N. Y. Med. J., Dec. 8. [5] Med. Chron., May. [6] Med. Chron., May. [7] Ther. Gazette, May. [8] N. Y. Med. J., Jan. 26. [9] Am. J. Obstet., Nov. 1894. [10] Lanc., Dec. 8. [11] B. M. J., Nov. 3. [12] B. M. J., Dec. 15. [13] Lanc., Dec. 27. [14] Lanc., June 1, 1895. [15] Med. Week., March 8. [16] Ther. Gazette, Dec. 15, 1894. [17] B. M. J., Dec. 1. [18] B. M. J., Nov. 24. [19] B. M. J., Jan. 5, 1895. [20] Med. Chron., Ap. [21] Les N. Remedés, Jan. 8. [22] Ther. Gazette, Nov. 15. [23] Med. Week., Jan. 25, 1895.

